# Overcoming Biological Barriers With Block Copolymers-Based Self-Assembled Nanocarriers. Recent Advances in Delivery of Anticancer Therapeutics

**DOI:** 10.3389/fphar.2020.593197

**Published:** 2020-11-30

**Authors:** Jazmin Torres, Namdev Dhas, Marcela Longhi, Mónica C. García

**Affiliations:** ^1^ Facultad de Ciencias Químicas, Universidad Nacional de Córdoba, Córdoba, Argentina; ^2^ Department of Pharmaceutics, Institute of Pharmacy, Nirma University, Ahmedabad, India; ^3^ Departamento de Ciencias Farmacéuticas, Facultad de Ciencias Químicas, Universidad Nacional de Córdoba, Córdoba, Argentina; ^4^ Unidad de Investigación y Desarrollo en Tecnología Farmacéutica (UNITEFA), Consejo Nacional de Investigaciones Científicas y Técnicas (CONICET), Córdoba, Argentina

**Keywords:** amphiphilic block copolymers, cell uptake, intracellular trafficking, stimuli-responsive nanocarriers, drug delivery, nanomedicine, tumor microenvironment barriers

## Abstract

Cancer is one of the most common life-threatening illness and it is the world’s second largest cause of death. Chemotherapeutic anticancer drugs have many disadvantages, which led to the need to develop novel strategies to overcome these shortcomings. Moreover, tumors are heterogenous in nature and there are various biological barriers that assist in treatment reisistance. In this sense, nanotechnology has provided new strategies for delivery of anticancer therapeutics. Recently, delivery platforms for overcoming biological barriers raised by tumor cells and tumor-bearing hosts have been reported. Among them, amphiphilic block copolymers (ABC)-based self-assembled nanocarriers have attracted researchers worldwide owing to their unique properties. In this work, we addressed different biological barriers for effective cancer treatment along with several strategies to overcome them by using ABC‐based self-assembled nanostructures, with special emphasis in those that have the ability to act as responsive nanocarriers to internal or external environmental clues to trigger release of the payload. These nanocarriers have shown promising properties to revolutionize cancer treatment and diagnosis, but there are still challenges for their successful translation to clinical applications.

## Introduction

Cancer is the world’s second largest cause of death, endangering tens of millions of people. Albeit the substantial development of significant drugs has advanced; their delivery is far from satisfactory (Zhou et al., 2020). Moreover, anticancer drugs have many disadvantages (undesirable biodistribution, low speciﬁcity, limited targeting, inefﬁcient cellular uptake, produce side/toxic effects) (Yin et al., 2016; Avramović et al., 2020). These limitations have prompted the development of novel strategies to overcome these shortcomings. Advanced nanotechnology has provided new approaches for efficient delivery of anticancer therapeutics and supposed to be a promising tool against this illness (Chan, 2017). Nevertheless, considering the relatively few nanomedicine choices available in clinical trials, this promise has apparently fizzled (Huo et al., 2019). Some failures originate from safety issues respect to long term exposure to engineered materials despite the advancements made to understand their nanotoxicity (Yan et al., 2019). Also, tumor multifaceted nature clarifies why most nanomedicines have not prevailing in clinical trials (Kievit and Zhang, 2011; Ernsting et al., 2013). It has been proposed that tumors are heterogenous in nature, possessing various biological barriers that assist in treatment resistance. Recent observations into the tumor niche indicate that many obstacles still exist and insufficient attention is given to their biological consequences when novel nanomedicines are designed (Huo et al., 2019). As a result, in the last decade new delivery platforms to overcome biological barriers caused by tumor cells and tumor-bearing hosts have been evaluated.

Many delivery systems on nanoscale with significant achievements have been reported to date. Among them, amphiphilic block copolymers (ABC)-based self-assembled nanocarriers (SAN) for delivery of anticancer therapeutics have attracted researchers worldwide owing to their unique chemical and physical properties, improved biocompatibility, tunable compositions, extended blood circulation, and facile functionalization (Yin et al., 2016; García et al., 2018; García and Quiroz, 2018; García, 2019b). With the advancements in polymer chemistry, different ABC with preferred number and monomer type, each having different hydrophobic and hydrophilic properties have been synthetized (Yorulmaz Avsar et al., 2019). By tailoring monomer combinations, SAN with upgraded properties to overcome biological barriers can be obtained. ABC can self-assemble into distinctive nanostructural arrangements among which micelles and more recently polymer vesicles (also termed as polymersomes) are the most reported (Yin et al., 2016; García and Quiroz, 2018; García et al., 2018). Inspired by their promising properties, nanomedicines could be intended to successfully mimic, improve or interact with these cross-talks for improving cancer therapy.

In this review, we addressed different biological barriers for numerous anticancer therapeutic moieties for effective cancer treatment along with several strategies to overcome them by using ABC-based SAN, with special emphasis in those that have the ability to act as responsive nanocarriers to internal or external environmental clues, producing on-demand triggered release of the payload.

## Biological Barriers and Strategies to Overcome Barriers for Effective Delivery of Anticancer Therapeutic

**FIGURE 1:**
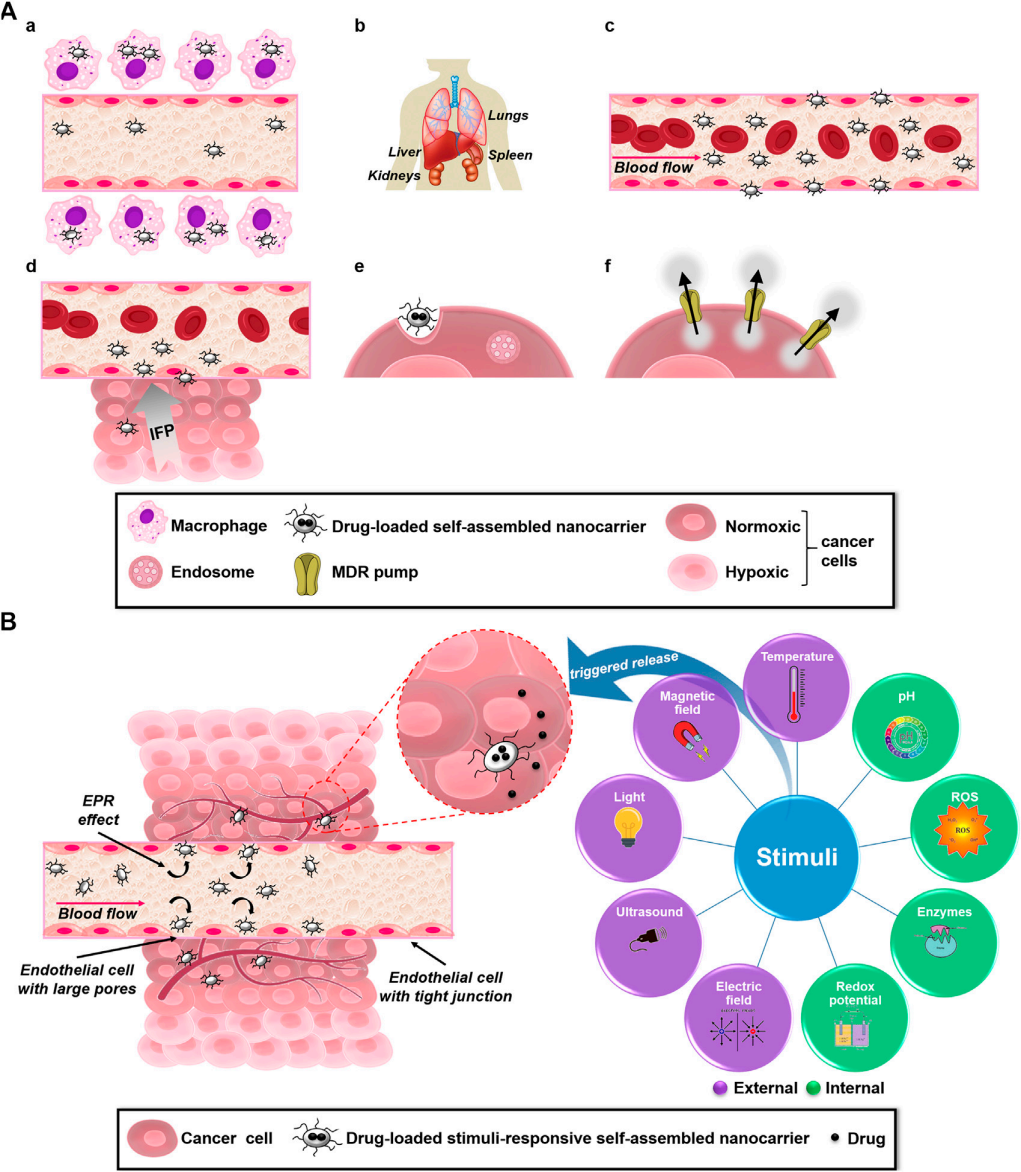
**(A)** Main and sequential biological barriers (after intravenous administration) faced to therapeutic delivery nanocarriers for effective cancer therapy: a) they may undergo opsonization and uptake by macrophages of the mononuclear phagocytic system; b) nonspecific distribution that lead accumulation of nanocarriers in other healthy organs such as spleen, livers and lungs, and extravasation and renal clearance faced by nanocarriers smaller than 5–6 nm; c) flow in blood vessels and endothelial surfaces; d) cancer microenviroment, including interstitial fluid pressure (IFP); e) cellular internalization and endosomal escape; and f) upon entry into tumor cells, multidrug resistant (MDR) system, including drug efflux pumps that remove anticancer therapeutics from the tumor cell. **(B)** Schematic representation of tumor environment and summary of internal and external stimuli for triggered delivery of anticancer therapeutics from self-assembled nanocarriers.

Cancer nanomedicine efficacy is measured primarily by how much drug can reach to the tumor-site. The properties of the biological barriers are diverse and represent both a challenge as well as an opportunity to develop tailor-made drug delivery system to effectively reach the target site. Distinct biological barriers and their peculiarities (Figure 1A), and strategies to overcome them are discussed in the following section.

### Opsonization/Sequestration by the Mononuclear Phagocytic System

After intravenous administration, nanocarriers are exposed to a several sequential hurdles that must be overcome in order to efficiently reach therapeutic levels of drugs at disease sites, avoiding nonspecific uptake in healthy organs (Blanco et al., 2015; Chevalier et al., 2017). As per the literature, when nanocarriers are intravenously administered, they can interact with thousands of proteins. Biological media consists of numerous active biomolecules such as lipids, nucleic acids, proteins, and blood plasma contains roughly 3,700 identified proteins (Lazarovits et al., 2015; Chen et al., 2017). Protein corona effect i.e. due to the presence of charge on nanocarriers can result into interaction with oppositely charged serum protein. Thus, nanocarriers or the targeting moiety decorated over nanocarriers get buried in protein corona resulting into prevention of nanocarriers’s interaction with the targeted receptor in cancer cells (Tonigold et al., 2018). Opsonins are key proteins in the blood serum that promotes elimination of vulnerable nanocarriers by marking them off for an immune response. The major part of opsonized nanocarriers are cleared within seconds by a receptor-mediated mechanism because of the phagocytic cells in the liver, lymph nodes, and spleen of the mononuclear phagocyte system, contributing to nonspecific distribution of nanocarriers in healthy organs (Blanco et al., 2015; Chevalier et al., 2017).

One most established strategy to mitigate the effect of protein corona involves the nanocarrier functionalization with amphiphilic or hydrophilic polymers, such as poly (ethylene glycol) (PEG) (Bros et al., 2018). PEG forms strong adhered hydration layer, which sterically avoid the interaction/adsorption of serum protein (Huo et al., 2019). PEG-decorated nanocarrriers reduce their recognition by the components of the mononuclear phagocytic system, increasing their blood circulation time (Xiong et al., 2009; Naeye et al., 2010). Zwitterionic ligands (i.e., sulfobetaine and carboxybetaine) have also been explored as antifouling agents (Kane et al., 2003; Tsai et al., 2004; Shao and Jiang, 2014; Li et al., 2018). These strategies are different since stacking of PEG chains is driven by Van der Waals forces and hydrophobic interactions, resulting into hard to form an ideal self-assembled monolayer; whereas zwitterionic ligands exhibit strong electrostatic interactions within their molecules, resulting into dense packing and minimizing protein adsorption on their surface. Additionally, charges on zwitterionic polymers can prompt strong hydration which retards protein adsorption (Huo et al., 2019).

### Vascular Flow

Blood vessels at tumor site develop gaps between endothelial cells during angiogenesis process through which nanocarriers can easily pass for reaching tumors by EPR effect. However, this effect is now in dispute in clinical trials since nanocarriers have shown moderate therapeutic efficacy. It could have happened due to heterogeneity of EPR effect in humans. For instance, mouse xenograft models develop tumor very fast and endothelial cells in blood vessels tend to present many fenestrations. Aversely, tumors in humans develop very slowly and not all tumor vessels develop inter-endothelial gaps (Li J.-X. et al., 2020). Recently, Matsumoto *et al.* demonstrated that tumor blood vessels form transient opening and closing as they dynamically generate vascular bursts, resulting into eruption and vigorous fluid outflow into tumor interstitial space which ultimately lead to nanocarrier extravasation (Matsumoto et al., 2016). In contrast, Sindhwani *et al.* proposed that extravasation takes place *via* interconnected vesiculovacuolar organelles (*trans*-endothelial pathway), revealing that 97% of nanocarrier extravasation occurred through this pathway (Sindhwani et al., 2020).

Different strategies to overcome tumor vascular barrier and improve accumulation of nanocarriers at tumor site can be use. 1) Enhancing tumor vascular permeability: a) physical stimulation viz. hyperthermia (Liu et al., 2018), ultrasound (Ho et al., 2016) and radiation (Potchen et al., 1972) allow increasing the local vascular permeability through which nanocarriers can efficiently pass (Ding et al., 2019). b) Use of disruptive agents (i.e., nitric acid) (Qin and Gao, 2019). It has been demonstrated that nitric acid can dilate blood vessels, elevate blood flow and also open tight junctions between endothelial cells (Maeda et al., 2013). 2) Targeting tumor vasculature: tumor cells overexpress distinctive biomarkers compared to normal endothelial cells; thus, active targeting of nanocarriers to tumor endothelial cells can overcome this barrier (Li J.-X. et al., 2020).

### Tumor Microenvironment

After crossing the vascular barrier, nanocarriers come across tumor microenvironment barrier composed by dense extracellular matrix (ECM), high interstitial fluid pressure (IFP) and stromal cells (Ding et al., 2019). ECM is a complex molecular structure mainly consisting of collagen network, proteoglycan, glycosaminoglycan, microfiber elastin and other polysaccharides with distinct biochemical and physical properties (Koláčná et al., 2007; Lu et al., 2012). Nanocarriers cannot cross this barrier because of the following reasons: 1) densely packed gel-like structure of tumor ECM increased lysyloxidase levels and the presence of integrin receptors with high viscosity (Barua and Mitragotri, 2014); 2) ECM has pores with 40 nm in size; thus, nanocarriers larger than 60 nm exhibit difficulties to cross this barrier (Zhang et al., 2018); 3) cancer cell density compresses the ECM and increases the IFP (solid tumors = 5–40 mmHg, pancreatic tumor = 75–130 mmHg whereas normal IFP = 0–3 mmHg) (Miao et al., 2015). Moreover, stromal cells that consist of cancer-associated fibroblasts (CAF) are mostly found in tumor microenvironment. CAF produce dense ECM, facilitate the tumor growth, and angiogenesis as they secrete cytokines viz. interleukin (IL)-6, hepatocyte growth factor and vascular endothelial growth factor, which hinder delivery of nanocarriers to target site (Kalluri, 2016). Also, CAF may express receptors similar to tumor cells to which nanocarriers can be targeted, leading to off target distribution (Sherman et al., 2014; Miao et al., 2016).

For overcoming this barrier, physical methods, such as ultrasound, phototherapy and hyperthermia, can be used since they disrupt ECM. However, these methods can affect normal healthy nearby tissues (Eggen et al., 2014). Enzymes viz. hyaluronidase (Zhou et al., 2016) and collagenase (Kuhn et al., 2006) constitute another strategy. They can effectively degrade the hyaluronan in tumor ECM and reduce IFP, enhancing nanocarrier penetration. microenvironment, such as stimuli-responsive (García, 2019b) or size-switching (Cabral et al., 2011) delivery platforms, are other strategies for enhancing cancer therapy. Size-switching nanocarriers are those with large particle size in blood circulation, which when become in contact with tumor tissue switch their size to small to effectively extravasate, leading to deep penetration (Cabral et al., 2011). Because of the advancements in the development of stimuli-responsive nanocarriers, in *Stimuli-Responsive Self-Assembled Nanocarriers Nanocarriers* they are described.

Furthermore, it has to be addressed that an additional important feature of tumor microenvironment is the overall immunosuppressive microenvironment because of the presence of numerous immune cells viz. dendritic cells (DC), tumor associated macrophages (TAM, including M1-immunostimulatory and M2-immunosuppressive phenotypes), natural killer (NK) cells, B and T lymphocytes, which have complex and diverse roles (Zhou et al., 2020). M2 phenotype is primarily triggered in tumors in response to hypoxic condition and facilitate metastasis, angiogenesis and decrease in immune response of T cells. Targeting TAM and switching from M2 to M1 phenotype, which reverse the immune suppression, can be an efficient anticancer strategy for preventing tumor progression (Zhang B. et al., 2017). Myeloid-derived suppressor cells and regulatory T-cells are also responsible for immune suppression (Munn and Bronte, 2016). They generate large amount of IL-10, arginine-1 and NO; and IL-10 and transforming growth factor (TGF)-β, respectively, which directly suppresses T cell function and release granzyme and perforin to destroy effector T cells, resulting into immunosuppression (Talmadge and Gabrilovich, 2013). Additionally, prostaglandin E-2, programmed death-1 molecule, TGF-β and IL-10 could suppress the antigen cross presenting ability of DC. Tumor cells also downregulate NK cells to prevent exposure of tumor necrosis factor-related apoptosis inducing ligand (Waldhauer and Steinle, 2008). Due to decrease trafficking in tumor sites (including downregulation of cluster of differentiation (CD) 62L on CD8^+^ T cells, the adhesion molecules VCAM-1 and ICAM-1, and IL-12) and lack of activation caused by the aforementioned immunosuppressive tumor microenvironment, the CD8^+^ T cells are not able to exhibit an inhibitory response against tumors (Buoncervello et al., 2019). Thus, targeting immune cells or immune related specific molecules could reshape the immune microenvironment and increase the treatment efficacy.

### Cellular Membrane

This membrane not only supplies nutrients to cells but also acts as one of the barrier for cellular internalization of nanocarriers (Choudhury et al., 2019), which is mostly based on their physicochemical properties and the protein corona that coated them. Therapeutic moieties with low molecular weight and lipophilic nature cross the membrane lipid bilayer by passive diffusion while molecules with higher molecular weight require active transportation (Blanco et al., 2015). Furthermore, numerous factors of nanocarriers (particle size, shape, surface charge, and hydrophobicity) affect cellular internalization. The particle size determines the type of endocytosis through cell membrane. Shape structure other than sphere, viz. anisotropy and original orientation are critical for nanocarrier uptake through cellular membrane. Also, this parameter can have dramatic effects on targeting, circulation, internalization, immune cell association, and adhesion (Champion et al., 2007; Sharma et al., 2010; Truong et al., 2015). Mechanical properties can also affect cell adhesion/penetration. Enhanced permeability and retention (EPR) effect is favored if nanocarriers exhibit good flexibility. Their elasticity increases their chances of penetration between endothelial cancer cells and also, in targeted drug release, nanocarriers’ flexibility is a key factor in the interaction with cellular receptor-mediated endocytosis (Anselmo et al., 2015).

### Multidrug Resistant System

MDR is a state of resistance toward structurally and/or functionally different therapeutic moieties and grouped into five classes: 1) increase efflux of drugs, primarily *via* adenosine triphosphate-driven extrusion pumps of adenosine triphosphate-binding cassette superfamily, such as breast cancer resistance proteins, MDR-associated protein 1, and P-glycoprotein, which form a unique defense system against anticancer drugs and exo- and endotoxins, significantly reducing intracellular concentration of drugs or endogenous toxins; 2) decrease influx of drugs; 3) DNA repair activation; 4) inactivation of apoptosis pathways with parallel activation of antiapoptotic cellular defensive compartments; 5) metabolic modification and detoxification.

MDR hinders to reach tumor-site by drug in the concentration and at the time required. Hence, only a fraction of dose remains into the cancer cells, while the remaining is removed by MDR (Choudhury et al., 2019; Huo et al., 2019).

## Amphiphilic Block Copolymers and Self-Assembled Nanocarriers

Self-assembly process refers to the spontaneous association of ordered and well-organized systems, which occurs under kinetic and thermodynamic conditions, allowing local and specific molecular interactions to form stable and perfectly structured aggregates. Electrostatic or hydrophobic interactions, hydrogen bonding, π-π aromatic-stacking, and van der Waals forces contribute to keep the molecules organized, achieving the minimal energy in the nanocarriers (Yadav et al., 2020).

SAN are easy to prepare, flexible and low-cost materials that have gained popularity for biomedical application in cancer (Berbezier and De Crescenzi, 2015; García and Quiroz, 2018). Moreover, their preparation involves generally large-scale techniques. Top-down and bottom-up approaches can be employed. While the former involves the transformation of a block of matter into a structured and organized nanostructure, the later requires the assembly of basic units/monomers for arriving at the nanocarrier structures through non-covalent interactions. Regardless of being weaks, altogether these interactions become the main force of the assembly process. Even though it is important to consider the balance between attractive driving, repulsive and directional forces (Yadav et al., 2020), simple but effective bottom-up approach is the most employed method. By tailoring experimental parameters different types of nanocarriers with distinctive morphologies and high performance for therapeutic purposes can be readily and selectively produced (Fratoddi et al., 2016; García and Quiroz, 2018). SAN represent a promising tool for targeted drug delivery, especially in personalized cancer treatments. They allow a site-directed delivery of anticancer therapeutics, increasing treatment efficacy and safety by modifying their physicochemical characteristics (i.e., size, shape, solubility, permeability) (García, 2019b; Yadav et al., 2020).

## Stimuli-Responsive Self-Assembled Nanocarriers

Even though passive and active targeting strategies contribute to intracellular accumulation of anticancer therapeutics, nanocarrier delivery performance can be improved by incorporating appropriate trigger responsiveness. Cutting-edge and emerging research focuses comprise the development of SAN since they can recognize some environmental clue, internal and/or external stimuli (Figure 1B), inducing physical/chemical changes and triggering cargo release in dose-, spatial-, and temporal-controlled manners (García, 2019b). Table 1 describes several examples of stimuli-responsive SAN and their application in cancer therapy.

**TABLE 1:** Amphiphilic block copolymers and stimuli-responsive self-assembly-based delivery systems for cancer therapy.

Stimulus	Type of nanocarrier	Nanocarrier *b*uilding *b*locks	Therapeutic agent loaded/Cargo	Type of tumor	Stage of development	Ref
Light	Micelles	Poly(AzoMA)-*b*-poly(β-AcGalEtMA)	Nile red	Melanoma	*In vitro* (A375 cells)	Pearson et al. (2015)
Light	Photochromic polymersomes	PEO-*b*-PSPA	DAPI	Cervical cancer	*In vitro* (HeLa cells	Wang X. et al. (2015)
Light	Polymersomes	CB[8]-MMV-*b*-TBA-Azo	Rhodamine B, DOX and 5(6)-carboxyfluorescein	Breast, lung and prostate cancer	*In vitro* (A549, MDA-MB-231, and PC-3 cancer cells; HUVEC and L-O2 normal cells)	Hu et al. (2018)
Light/pH	Multi-compartment vesicles and complex micelles	β-CD-acylhydrazone-DOX and Azo-PDMA-FA	DOX	Breast cancer	*In vitro* (MCF-7 cells)	Bai et al. (2018)
Light/pH	PIC micelles	PDMNBMA-*b*-PCBMA	FITC and BSA	Lung cancer	*In vitro* (A549 cells)	Jin et al. (2014)
Light (NIR)/GSH	Micelles	PCL-SS-BPLP and biotin-PEG-cypate	DOX and cypate	Liver and lung cancer	*In vitro* (HepG2 cell) and *in vivo* (C57BL/6 mice, Lewis lung cancer)	Zhang et al. (2019)
Light (NIR)/ROS	Polymersomes	PPS -*b*-PEG	ZnPc and DOX	Melanoma	*In vitro* (A375 cells)	Tang et al. (2020)
Magnetic field	Polymersomes	PTMC-*b*-PGA	γ-Fe_2_O_3_ and DOX	Cervical cancer	*In vitro* (HeLa cells)	Oliveira et al. (2013)
Magnetic field	Asymmetrical vesicles	R-PGA-*b*-PCL [R is FA or DTPA]	DOX and gadolinium [Gd(III)]	Liver cancer	*In vitro* (SMMC-7721 cancer cells and (L02) normal cells)	Liu Q. et al. (2015)
Magnetic field	Micelles	PNIPAM-*b*-PCL-*b*-PNIPAM	Fe_3_O_4_ nanoparticles and paclitaxel	Breast cancer	*In vitro* (MCF-7 cells)	Pourjavadi et al. (2019)
Temperature	Micelles	PFAAM-*b*- PFPAM	Paclitaxel	Liver cancer	*In vitro* (A549 and Bel 7402 cells)	Chen et al. (2013)
Temperature	Micelles	PNIPAM-*b*-HTPB-*b*-PNIAM	Campothecin	Breast cancer	*In vitro* (MDA-MB231)	Luo et al. (2014)
Temperature	Polymersomes	PVCL-*b*-PDMS-*b*-PVCL	DOX	Lung cancer	*In vitro* (A549 cells)	Liu F. et al. (2015)
Temperature	Micelles	PE-PCL-*b*-PNIPAM-FA and PE-PCL-*b*-PNVCL-FA	DOX	Glioblastoma	*In vitro* (C6 glioma cells) and *in vivo* (C6 glioma tumor rat model)	Panja et al. (2016)
Temperature	Polymersomes	PMVC-PVPON	DOX	—	*In vivo* (C57BL/6J mice)	Kozlovskaya et al. (2019)
Temperature/pH	Micelles	PNIPAM-*co*-PCL and PNIPAM- *co*-N,N-dimethylacrylamide-*b*-lacitde	Adriamycin	Stomach cancer	*In vitro* (N-87 cells)	Li W. et al. (2011)
Ultrasound	Micelles	Plurconic P123/Plurconic F127	Curcumin	Breast cancer	*In vitro* (MDA-MB-231 and 4T1 cells) and *in vivo* (BALB/c mice, 4T1)	Wu et al. (2017)
Ultrasound/pH	Polymersomes	PEO-*b*-P(DEA-*stat*-MEMA)	DOX	Cervical cancer	*In vitro* (HeLa cells) and *in vivo* (BALB/c nude mice, HeLa cells)	Wei P. et al. (2020)
pH	Polymersomes	PLL-CA/PEG-DOX	DOX	Breast cancer	*In vitro* (MCF-7 cells)	Zhu et al. (2012)
pH	Micelles	mPEG-*b*-P(CL-co-DCL)	DOX	Liver cancer	*In vitro* (HepG2 cells)	Deng et al. (2014)
pH	Micelles	PGA-*b*-PLA	DOX	Melanoma	*In vitro* (A375 cells) and *in vivo* (Balb/c nude mice, A375 cells)	Wang Q.-M. et al. (2014)
pH	Polymersomes	PEG-*b*-PTTAMA	Nile red and DOX	Cervical cancer	*In vitro* (HeLa cells)	Wang L. et al. (2015)
pH	Micelles	PEG-*b*-PAU-*b*-PEG	DOX	Breast cancer and leukemia	*In vitro* (MCF-7/ADR and RAW 264.7 cells)	Huang et al. (2015)
pH	Chimeric polymersomes	Acupa-PEG-PTMBPEC-PSAC	BSA, cytochrome C, and granzyme B	Prostate cancer	*In vitro* (LNCaP and PC-3 cells) and *in vivo* (nude mice)	Li et al. (2015)
pH	PIC micelles	PEG-*b*-PLL	DOX	Liver cancer	*In vitro* (HepG2 cells) and *in vivo* (xenograft human HepG2 hepatoma-bearing nude mouse)	Zheng et al. (2020)
pH	Micelle	Dex-*g*-(DOX + BTZ)/cRGD (polysaccharide-di-drugs conjugate)	DOX and BTZ	Melanoma	*In vitro* (B16F10 cells) and *in vivo* (melanoma-allografted BALB/c mice)	Li D. et al. (2020)
Enzyrme	Polymersomes	GFLGF peptide-containing mPEG-*b*-PDLLA	Fluorescein	Breast cancer	*In vitro* (SKBR3 cells)	Lee et al. (2011)
Enzyrme	Polymersomes	Dex-PDP or DEX-CAR	Rhodamine-B and camptothecin	—	*In vitro* (MEFs cells)	Pramod et al. (2012)
Enzyrme	Polymersomes	Dex-PDP or DEX-CAR	DOX and camptothecin	Breast and colon cancer	*In vitro* (MCF7 and DLD1, cells)	Pramod et al. (2014)
Enzyme	Nanoassemblies	mPEG-Pep-PCL and FA-PEG-PCL	Camptothecin	Melanoma	*In vitro* (B16 cells) and *in vivo* (ICR mice, B16 cells)	Yu et al. (2015)
Enzyme	Nanoassemblies	PCL-*b*-carboxylic PCL	DOX	Breast and cervical cancer	*In vitro* (MCF7 and HeLa cells)	Malhotra et al. (2016)
Enzyrme	Micelles	PEG-GPLGVRGDG-P(BLA-co-Asp)	DOX	Fibrosarcoma	*In vitro* (HT1080 cells)	Ke et al. (2016)
Enzyme	Nanoassemblies	l-Tyrosine Poly(ester-urethane)s	DOX and camptothecin	Cervical cancer	*In vitro* (HeLa and WT-MEF cells)	Aluri and Jayakannan (2017)
Enzyrme	Nanoassemblies	PEG- GFLG-GEM	Gemcitabine (GEM)	Breast cancer	*In vitro* (4T1 cells)	Zhang C. et al. (2017)
Enzyme/pH	Polymersomes	Dex-IM-PDP	DOX	Breast cancer	*In vitro* (MCF7 cells)	Pramod et al. (2015)
ROS	Polymersomes	PEG-*b*-PPS	Gardiquimod and ovalbumin	—	*In vitro* (mouse bone marrow-derived dendritic cells)	Scott et al. (2012)
ROS	Polymersomes	P[(HPMA) -*b*-(ROS1/2)]	DOX	Lymphoma	*In vitro* (EL4 T cells) and *in vivo* (C57BL/6J mice, EL4 T cells)	Jäger et al. (2020)
ROS/pH	Polymersomes	PEO-*b*-PNBMA	DOX and paclitaxel	Cervical cancer	*In vitro* (HeLa and RAW 264.7)	Deng et al. (2016)
GSH	Micelles	mPEG-SS-PzLL	DOX	Breast cancer	*In vitro* (MCF7 cells)	Wen et al. (2011)
GSH	Shell-detachable micelles	PCL-SS- PEEP	DOX	Breast cancer	*In vitro* (MCF-7/ADR)	Wang et al. (2011)
GSH	Polymersomes	PEG-*b*-PLL-SS-PCL	DOX and camptothecin	Squamous carcinoma	*In vitro* (SCC7 cells)	Thambi et al. (2012)
GSH	Different hierarchical nanoassemblies (spheres, large compound vesicles, smooth disks, and staggered lamellae)	PEG-*b*-PCPTM	Camptothecin	Liver and lung cancer	*In vitro* (HepG2 and A549 cells)	Hu et al. (2013)
GSH	Micelles	PCL-SS-PDMA and PCL-SS-PDMA/DNA	DOX and DNA	Cervical cancer and oral carcinoma	*In vitro* (HeLa, KB and CAL-27 cells)	Li et al. (2014)
GSH	Polymersomes	PEG–PAA– PDEA and PEG–PAA(SH)–PDEA	BSA and and cytochrome C	Breast and cervical cancer	*In vitro* (MCF-7 and HeLa cells)	Sun et al. (2014)
GSH	Polymersomes	cNGQ-PEG-P(TMC-DTC)	DOX	Lung cancer	*In vitro* (A549 cells) and *in vivo* (orthotopic A549 human lung cancer xenografts in nude mice)	Zou et al. (2016)
GSH	Micelles	mPEG-SS-paclitaxel and mPEG-SS-DOX conjugates	DOX and paclitaxel	Lung cancer and melanoma	*In vitro* (A549 and B16 cancer cells) and *in vivo* (B16 mouse melanoma model)	Zhao et al. (2017)
GSH	Polymersomes	FA-PCL-SS-PEG-SS-PCL	DOX and paclitaxel, P-glycoprotein inhibitor tariquidar	Breast cancer	*In vitro* (MCF-7/ADR cells)	Qin et al. (2018)
GSH	Polymersomes and micelles	PNIPAM-*b*-PDS-*b*-PNIPAM and PTEGMA-*b*-PDS-*b*-PTEGMA	DOX	Cervical cancer	*In vitro* (HeLa cells)	Bej et al. (2018)
GSH	Nanoassemblies	Xyl-SS-curcumin	5-Fluorouracil and curcumin	Colorectal cancer	*In vitro* (HT-29 and HCT-15 cells)	Kumar et al. (2020)
GSH	Polymersomes	TBP-PEG-P(TMC-DTC)	DOX	Colorectal cancer	*In vitro* (HCT-116 cells) and *in vivo* (Balb/c nude mice, HCT-116 cells)	Wei Y. et al. (2020)
GSH	Chimeric polymersomes	HA-RCP- granzyme B	Granzyme B	Multiple myeloma	*In vitro* (NALM-6, K562, MM1S, and LP1 cells) *In vivo* (nude mice, LP1 cells)	Zhong et al. (2020)

Acronyms’ details: AzoMA, 4-[4-[(4-Methoxyphenyl)azo]phenoxy]ethanol; β-AcGalEtMA, 2-(2,3,4,6-Tetra-O-acetyl-β-d-galactopyranosyl)ethyl methacrylate; DOX, doxorubicin; Azo-PDMA-FA, azobenzene-terminated poly(2-(dimethylamino)ethyl methacrylate); β-CD, β-cyclodextrin; PEG, poly(ethylene glycol); isoAZO/C18, 4-isobutyloxyazobenzene units (AZO) and hydrocarbon chains (C18); CB [8], cucurbit [8] uril; MMV, maleimide-modified methylviologen; PSPMA, poly(spiropyran ether methacrylate); PEO, poly(ethylene oxide); SPA, spiropyran (SP)-based monomer containing a carbamate linkage; DAPI, 4′,6-diamidino-2-phenylindole; PDMNBMA, poly(N,N-dimethyl-N-(2-(methacryloyloxy)ethyl)-N-((2-nitrobenzyl)oxy)-2-oxoethanaminium bromide); PCBMA, poly(carboxybetaine methacrylate); BSA, bovine serum albumin; PBC, poly(benzyl carbamate); PDMA, poly(N,N-dimethylacrylamide); PCL, poly(caprolactone), SS, disulfide bond; BPLP, biodegradable photoluminescent polymer; ZnPc, Zinc phthalocyanine photosensitizer; PPS, poly (propylene sulfide); PNIPAM, poly-N-isopropylacrylamide; PTMC, poly(trimethylene carbonate); PGA, poly(L-glutamic acid); P2VP, poly(2-vinylpyridine); FA, folic acid; DTPA, diethylenetriaminepentacetatic acid; CS, chitosan; P(tBA-co-AA), poly(t-butyl acrylate-co-acrylic acid); PFAAM, P(folate-allylamine-co-NIPA-co-acrylamide-co-octadecyl acrylate); PFPAM, and P(folate-PEG-acrylic acid-co-NIPA-co-acrylamide-co-octadecyl acrylate); HTPB, hydroxyl-terminated polybutadiene; PE, pentaerythritol; PNVCL, poly(N-vinylcaprolactam); PDMS, polydimethylsiloxane; PMVC, poly(3-methyl-N-vinylcaprolactam); PVPON, poly(N-vinylpyrrolidone); P(DEA-stat-MEMA), poly(2-(diethylamino)ethyl methacrylate)-stat-poly(methoxyethyl methacrylate); Fc, ferrocene; PLL, poly(l-lysine); P(CL-co-DCL), poly(ε-caprolactone-co-γ-dimethyl maleamidic acid; BTZ, bortezomib; Dex, dextran; cRGD, cyclo-(Arg-Gly-Asp-D-Phe-Lys) peptide; CA, cholate; PTTAMA, poly(2-((((5-methyl-2-(2,4,6-trimethoxyphenyl)-1,3-dioxan-5-yl)methoxy)carbonyl)amino)ethyl methacrylate); PAU, poly(acetal urethane); mPEG, methoxy PEG; PDLLA, poly(D,L-lactide); GFLG, glycyl phenylalanyl leucyl glycine tetra-peptide; PBLA, poly(β-benzyl l-aspartate); Pep, metalloproteinase-2 and metalloproteinase-9; PDP, Ethyl 2-(3-pentadecylphenoxy)acetate; CAR, 2-(3-pentadec-7-enyl)phenoxy)acetic acid; ROS monomer 1 and 2: 4- aminophenylboronic acid pinacol ester and 4- (hydroxymethyl)phenylboronic acid pinacol ester, respectively; P(HPMA), azide-terminated poly([N-(2-hydroxypropyl)]- methacrylamide); PzLL, poly(e-benzyloxycarbonyl-L-lysine); PEEP, poly(ethyl ethylene phosphate); Xyl, xylan; PCPTM, reduction-cleavable camptothecin prodrug monomer; PDEA, poly(2-(diethyl amino)ethyl methacrylate); P(TMC-DTC)), poly(trimethylene carbonate-co-dithiolane trimethylene carbonate); cNGQGEQc, cyclic peptide cNGQGEQc; PDS, poly(disulfide); PTEGMA, poly(triethyleneglycol)methylethermethacrylate; TBP, transferrin binding peptide CGGGHKYLRW; HA, hyaluronic acid.

### External Stimuli-Responsive Nanocarriers

As stated, different strategies to overcome biological barriers for delivery of anticancer therapeutics, such as hyperthermia (Liu et al., 2018), ultrasound (Ho et al., 2016) and radiation (Potchen et al., 1972) are used. Interestingly, they can be also explored in the design of SAN that can be remotely controlled for triggering cargo delivery.

#### Light-Responsive Nanocarriers

Light irradiation, with variable intensity and wavelength, is an easy and low-cost exogenous stimulus. At a specific time and location, the disassembly of nanocarriers can be induced upon exposure to certain wavelengths, such as ultraviolet, visible, and near-infrared (NIR) (Che and van Hest, 2016; Johnson and Preman, 2018; García, 2019b). Light-responsive SAN frequently incorporate ABC with photo-sensitive moieties viz. azobenzene (Blasco et al., 2013a; Blasco et al., 2013b; Blasco et al., 2014; Xia et al., 2014; Pearson et al., 2015; Bai et al., 2018; Hu et al., 2018), spiropyran (Wang B. et al., 2014; Wang X. et al., 2015; Kwangmettatam and Kudernac, 2018), *o*-nitrobenzyl (Jin et al., 2014; Liu et al., 2014; Yamamoto et al., 2019), chromophores (Hribar et al., 2011; Song et al., 2011; Yan et al., 2011; He et al., 2013; Lin et al., 2013; Ding et al., 2015; Tang et al., 2020). They behave as light-cleavable linkers or induce light-sensitive degradation or conformational changes (García, 2019b). Drug release may be controlled by adjusting three main parameters: light intensity, wavelength, and exposure time. NIR-responsive SAN have been widely studied for non-invasive and on-demand drug delivery therapy (García, 2019b; Li F. et al., 2020) since tissue and skin exhibit minimum absorbance in the range 650–900 nm (Yin et al., 2016; Johnson and Preman, 2018; García, 2019b). Light-responsive SAN have also been studied for photothermal/photodynamic cancer therapy. The incorporation of gold-based nanostructures into SAN is useful for hyperthermia-based therapy (Liao et al., 2015).

#### Magnetic-Responsive Nanocarriers

Magnetic-responsive SAN have been evaluated in cancer therapy and/or diagnosis for magnetically triggered cargo release, magnetic resonance imaging, hyperthermia and magnetic guidance (Che and van Hest, 2016; Thambi et al., 2016; Hu et al., 2017; García, 2019b). High penetration, ease of control, noninvasive nature, and absence of energy dissipation are their main characteristics (García, 2019b). Commonly, ferromagnetic, paramagnetic or superparamagnetic (i.e., magnetite and maghemite) nanoparticles are incorporated into self-assemblies (Lecommandoux et al., 2005; Krack et al., 2008; Mart et al., 2009; Kim et al., 2010; Yang et al., 2010; Hickey et al., 2011; Meeuwissen et al., 2011; Sanson et al., 2011; Oliveira et al., 2013; Roger et al., 2014; Van Rhee et al., 2014; Liu Q. et al., 2015).

#### Temperature-Responsive Nanocarriers

Temperature is the most widespread stimulus to trigger the specific responsiveness of SAN for applications in cancer nanomedicine (Mu et al., 2019). Local temperature is slightly higher in solid tumors than in normal tissues; hence, nanocarriers may accumulate into the tumor by adjusting the thermo-responsiveness of ABC (phase transition temperature: upper or lower critical solution temperatures, UCST and LCST, respectively) (Ward and Georgiou, 2011) to be between body and tumor temperature (Onaca et al., 2009; Thambi et al., 2016; García, 2019b). Poly-*N*-isopropylacrylamide (PNIPAM) as block, with LCST = 32 °C (Che and van Hest, 2016), has been widely evaluated in ABC-based SAN (Qin et al., 2006; Onaca et al., 2009; Xu et al., 2009; Moughton and O’Reilly, 2010; Li G. et al., 2011; Li W. et al., 2011; Chen et al., 2013; Luo et al., 2014; Che and van Hest, 2016; Panja et al., 2016; Hu et al., 2017; García, 2019b). Magnetic nanocarriers that incorporated PNIPAM have also been reported, exhibiting dual responsiveness as well as usefulness for hyperthermia therapy (Bixner et al., 2016). Despite plenty PNIPAM-based nanocarriers have been described so far, their translation to clinical applications is still controversial, since preclinical studies indicated systemic toxicity (Johnson and Preman, 2018). Alternatively, poly(N-vinylcaprolactam)-containing ABC have been evaluated (Liu F. et al., 2015; Kozlovskaya et al., 2019). Moreover, the wide variety of polymers and possible conjugations for synthetizing thermo-sensitive ABC allow broad horizons in the development of temperature-responsive SAN for cancer therapy.

#### Ultrasound-Responsive Nanocarriers

Ultrasound is a promising stimulus due to its easy administration, low-cost, and deep tissue penetration by tuning the frequency, duty cycles and time of exposure. It has been used as adjuvant in cancer treatment, behaving as a sensitizer to improve chemotherapy and overcome drug resistance (Thambi et al., 2016; Hu et al., 2017; García, 2019b). Ultrasound induces bubbles and air-containing assemblies can trigger drug release and ultrasound-targeted cancer imaging (Zhou et al., 2006).

#### Electric Field-Responsive Nanocarriers

Electric field- or voltage-responsive SAN have shown interesting properties as well. This stimulus may produce changes in charge or polarity of ABC, affecting their chemical composition or structure, and evoking disassembly with the subsequent cargo release (Yan et al., 2010; Jang et al., 2014; García, 2019b; Li F. et al., 2020). Further studies are still needed to better understand their usefulness for cancer therapy.

### Internal Stimuli-Responsive Nanocarriers

Endogenous stimuli-responsive SAN exploit the characteristics of tumor microenvironment, which are completely different from normal tissue physiology; thus, allowing cargo release in a programmed manner to specific intracellular stimuli viz. low pH, redox state, reactive oxygen species (ROS), and enzymes (García, 2019b).

#### pH-Responsive Nanocarriers

Extracellular pH of tumor is ∼6.5–7.2, whereas in normal tissues and other biological fluids is ∼7.4. pH is even lower in intracellular endosomes (5.5–5.0) and lysosomes (4.0–4.5). ABC with acid-cleavable bonds or ionizable groups are intended SAN to carry and control cargo release at the low pH in the tumor microenvironment (Rodríguez-Hernández and Lecommandoux, 2005; Zhan et al., 2011; Du et al., 2012; Li et al., 2012; Wang L. et al., 2015; García, 2019b). ABC based on hydrolysis-susceptible aliphatic polyesters, such as poly(lactic acid) or poly(e-caprolactone) as hydrophobic blocks, can be used for obtaining pH-sensitive SAN (Ahmed et al., 2006; Deng et al., 2014). Several acid-cleavable linkers (i.e., hydrazone, imine, ortho ester, and acetal) can also be used. ABC with weak acidic groups such as carboxylic or sulfonic acids (i.e., poly(acrylic acid), poly(methacrylic acid)) and/or weak basic groups such amines [i.e., poly (*β*-amino ester), poly(lysine), poly(histidine)] allow preparing pH-sensitive SAN, which can suffer alterations in conformation or solubility *via* ionization (Hu et al., 2017; ; García, 2019a; García, 2019b). These groups can be incorporated into the main or pendant chains of ABC providing to SAN with tunable degradation kinetics (Du and Armes, 2005; Zhu et al., 2012; Wang L. et al., 2015; Huang et al., 2015; Che and van Hest, 2016; Hu et al., 2017). Triblock-containing ABC have also reported in pH-responsive SAN for anticancer therapeutic delivery (Dan and Ghosh, 2013; Li et al., 2015). Moreover, polypeptide-based ABC that self-assemble into polymersomes, also called pepsomes, have been evaluated for cancer therapy (Chécot et al., 2002; Kukula et al., 2002; Rodríguez-Hernández and Lecommandoux, 2005; Sanson et al., 2009; Sanson et al., 2010; Hu et al., 2017). Oppositely charged ABC that contain a PEG block and an aniomer/catiomer block were reported as well (Anraku et al., 2010). Polymersomes based on them, termed as polyion complexes/PICsomes (Hu et al., 2017), have exhibited tunable membrane permeability and long blood circulation (Anraku et al., 2010).

#### Enzyme-Responsive Nanocarriers

Cancer can cause altered expressions of different enzymes (Thomas and Jeong, 2019). Specific enzymes viz. peptidases, elastase, thermolysin (Habraken et al., 2011), penicillin-G amidase (Harnoy et al., 2014)*,* lysosomal esterase (Pramod et al., 2014; Pramod et al., 2015; Malhotra et al., 2016) have been evaluated for triggering anticancer drug release from enzyme-sensitive SAN (Che and van Hest, 2016; Hu et al., 2017). ABC based on GFLG and GPLGVRGDG peptide sequence have also been used for cancer therapy since they can be cleaved by cathepsin B (Lee et al., 2011; Zhang C. et al., 2017) and metalloproteinase enzyme (Yu et al., 2015; Ke et al., 2016), respectively. Despite significant advances in enzyme-responsive SAN for delivery of anticancer therapeutics, this is a relatively new area of research and remains to be evaluated (Thambi et al., 2016).

#### Reactive Oxygen Species- and Redox-Responsive Nanocarriers

ROS (i.e., H_2_O_2_, ONOO^−^, HO·, O_2_^−^, and ^1^O_2_) (Yin et al., 2016; García, 2019b) are associated with cancer cell growth. They reflect a disruption of redox homeostasis because of either higher ROS production or lower ROS-scavenging capacity in cancer cells than normal cells (Trachootham et al., 2009). Abnormal redox states in tumors and the distinctive characteristics from their surroundings encouraged site-specific delivery of anticancer therapeutics from ROS-responsive SAN (Napoli et al., 2004; Scott et al., 2012; Hu et al., 2017; García, 2019b). Boronic esters exhibit ROS responsiveness for H_2_O_2_-induced degradation (Song et al., 2013; Deng et al., 2016; Greten and Grivennikov, 2019; Jäger et al., 2020). Moreover, selenium-(Ma et al., 2010) and tellurium-containing (Wang et al., 2016) ABC are emergent materials for developing ROS-responsive SAN; however, their toxicity after degradation needs to be further studied (Cao et al., 2015). In addition, extracellular environments, such as body fluids and cell surface, have a lower glutathione (GSH) concentration (2–20 µM) than cytosol and nuclei (10 mM). Particularly, redox potential of cancer cells is 100- to 1,000-fold higher than other normal cells; hence, triggered intracellular delivery of anticancer therapeutics in this reductive environment can be achieved (Che and van Hest, 2016; García, 2019b). The incorporation of disulfide bonds in the middle/side chain of ABC or in a cross-linker provide redox responsiveness to SAN. Tumor-relevant GSH concentration cleavages these bonds, inducing nanocarrier disassembly and cargo release into cancer cells (Wen et al., 2011; Thambi et al., 2012; Hu et al., 2013; Jia et al., 2014; Li et al., 2014; Sun et al., 2014; Wen and Li, 2014; Che and van Hest, 2016; Thambi et al., 2016; Zou et al., 2016; Hu et al., 2017; Zhao et al., 2017; Bej et al., 2018; Qin et al., 2018; García, 2019b; Kumar et al., 2020; Wei Y. et al., 2020; Zhong et al., 2020).

## Concluding Remarks and Future Trends

Cancer treatment mainly relies on the use of anticancer drugs; however, their disadvantages negatively impact in therapeutic success. Besides, to reach the tumor-site they need overcome different biological barriers. However, these barriers can be overcome by developing well-designed nanocarriers. Several efforts have been made to comprehensively understand the ability of ABC to self-assemble into nanocarriers and disassemble depending on different environmental stimuli. Representative examples of ABC-based stimuli-responsive SAN were reviewed and their applications for delivery of anticancer therapeutics were highlighted. As stated, the incorporation of stimuli-triggered responsiveness allows them recognizing changes in external/internal environment, inducing on-demand release behavior in spatial-, temporal-, and dose-controlled fashions. Even though these nanocarriers have shown promising properties to revolutionize cancer therapy and diagnosis, there are still challenges for their successful translation to clinical applications. Their biocompatibility, long-term toxicity and immunogenicity need be more studied for establish their safety profile, and advanced *in vivo* studies are also required to better understand nanocarrier-organism interaction. Considering scalable/reproducible manufacturing process, significant efforts in design, synthesis, and optimization of ABC and their self-assemblies are needed.

## Author Contributions

MG conceived and proposed the idea. MG compiled the manuscript with support from JT, ND, ML.

## Conflict of Interest

The authors declare that the research was conducted in the absence of any commercial or financial relationships that could be construed as a potential conflict of interest.
